# Step Detection Accuracy and Energy Expenditure Estimation at Different Speeds by Three Accelerometers in a Controlled Environment in Overweight/Obese Subjects

**DOI:** 10.3390/jcm11123267

**Published:** 2022-06-07

**Authors:** Ville Stenbäck, Juhani Leppäluoto, Rosanna Juustila, Laura Niiranen, Dominique Gagnon, Mikko Tulppo, Karl-Heinz Herzig

**Affiliations:** 1 Research Unit of Biomedicine, Medical Research Center, Faculty of Medicine, University of Oulu, Oulu University Hospital, 90220 Oulu, Finland; ville.stenback@oulu.fi (V.S.); juhani.leppaluoto@oulu.fi (J.L.); rosanna.juustila@student.oulu.fi (R.J.); laura.niiranen@oulu.fi (L.N.); mikko.tulppo@oulu.fi (M.T.); 2 Helsinki Clinic for Sports and Exercise Medicine, Foundation for Sports and Exercise Medicine, 00550 Helsinki, Finland; ddgagnon@laurentian.ca; 3 Department of Sports and Exercise Medicine, Clinicum, University of Helsinki, 00014 Helsinki, Finland; 4 School of Kinesiology and Health Sciences, Laurentian University, Sudbury, ON P3E 2C6, Canada; 5 Center for Research in Occupational Safety and Health, Laurentian University, Sudbury, ON P3E 2C6, Canada; 6 Department of Pediatric Gastroenterology and Metabolic Diseases, Poznan University of Medical Sciences, 61-701 Poznan, Poland

**Keywords:** accelerometry, step detection, physical activity, energy expenditure, overweight

## Abstract

Our aim was to compare three research-grade accelerometers for their accuracy in step detection and energy expenditure (EE) estimation in a laboratory setting, at different speeds, especially in overweight/obese participants. Forty-eight overweight/obese subjects participated. Participants performed an exercise routine on a treadmill with six different speeds (1.5, 3, 4.5, 6, 7.5, and 9 km/h) for 4 min each. The exercise was recorded on video and subjects wore three accelerometers during the exercise: Sartorio Xelometer (SX, hip), activPAL (AP, thigh), and ActiGraph GT3X (AG, hip), and energy expenditure (EE) was estimated using indirect calorimetry for comparisons. For step detection, speed-wise mean absolute percentage errors for the SX ranged between 9.73–2.26, 6.39–0.95 for the AP, and 88.69–2.63 for the AG. The activPALs step detection was the most accurate. For EE estimation, the ranges were 21.41–15.15 for the SX, 57.38–12.36 for the AP, and 59.45–28.92 for the AG. All EE estimation errors were due to underestimation. All three devices were accurate in detecting steps when speed exceeded 4 km/h and inaccurate in EE estimation regardless of speed. Our results will guide users to recognize the differences, weaknesses, and strengths of the accelerometer devices and their algorithms.

## 1. Introduction

Obesity is one of the greatest threats to our health and wellbeing worldwide. In 2016, 1.9 billion (39%) adults were considered overweight, of which 650 million (13%) were obese [1]. Obesity is directly linked to disorders such as hypertension, type II diabetes, and cardiovascular disease, which can lead to further chronic disabilities [2]. In the USA alone, the costs of obesity for society are estimated to be USD 1.72 trillion yearly or 9.3% of their gross domestic product [3]. In Germany, the estimated direct and indirect costs are estimated at EUR 63.04 billion yearly or 1.87% of its gross domestic product [4]. These numbers are expected to climb since the prevalence of obesity is continuing to increase [5]. Proper diet and physical activity (PA) are the two most important strategies for weight loss and maintenance for the majority of patients. In addition, PA does not need additional financial resources and could be applied everywhere worldwide. The 2020 WHO Physical Activity Guidelines provide information on the health benefits of physical activity: Most adults should complete at least 150 min a week of moderate physical activity and muscle-strengthening exercises two times a week, or 75 min of vigorous physical activity [6]. Unfortunately, a large proportion of adults do not attain the level of the recommended physical activity.

To accurately assess the effects of PA on populations and create personalized recommendations, more reliable and objective tools are needed. The current recommendations are still largely based on self-reported measures, which include, e.g., asking for information on time used for leisure, household, and transportation activities. Accelerometry is a commonly used objective method to measure PA, but multiple and significant considerations remain. Waist-worn accelerometers are more accurate than wrist-worn ones, data counting systems and the availability of raw data differ between devices, and differences in signal processing, step detection, and filtering exist as well [7,8,9]. The gait characteristics of the obese include, for example, slower speed and shorter stride length when compared to normal weight people [10]. For most overweight/obese and elderly people, the self-selected walking pace is 3 km/h or lower [11,12]; hence, these low speeds are important when using accelerometry with these subjects and evaluating health benefits. A maximal gait speed of 7 km/h was reported for elderly and elderly obese people in the Baltimore Longitudinal Study of Aging [13]. To capture the habitual PA via accelerometry in overweight, obese, and elderly populations, accurately measuring slow walking is of the utmost importance. The “Gold standard” technique for measuring energy expenditure (EE) is the double-labeled water method, which accurately measures the overall EE from a period longer than 3–4 days but is costly [14]. Direct calorimetry can also be used to measure EE but requires a thermally isolated chamber in which the subject is measured. Indirect calorimetry can be used to estimate EE from the use of O_2_ and the production of CO_2_ from the ventilation gasses. Furthermore, accelerometers can be used in EE estimation with or without heart rate measurement [15]. A recent review by Pisanu and colleagues states, that EE estimation with accelerometers in overweight and obese subjects is inaccurate [16]. In addition, an underestimation of EE during semi-structured activity protocol including, for example, household activities with the ActiGraph GT3X was observed to be 26% in overweight subjects [17]. Earlier, we showed in normal weight subjects that there are significant differences in the accelerometers’ accuracy at different speeds, with decreasing accuracy at speeds of 3 km/h or less [18]. Few studies have investigated the accuracy of research-grade accelerometers in a controlled environment for step detection and EE estimation in overweight and obese people, and importantly, none have included gait speeds initiated at 1.5 km/h, a gait speed we have previously observed in people at risk of T2D [19]. Feito and colleagues (2012) studied the effect of BMI class to step detection accuracy with hip-mounted accelerometers at three different speeds (2.4, 4.0, and 5.6 km/h) and found an error-%s of 20–60% at the lowest speed with no difference between the BMI-classes [20]. Error percentages in EE estimation have been shown to be 40–31% in overweight and obese subjects using the Freedson 1996 cut-off points with speeds starting from 4 km/h [21]. 

Our aim was to investigate the accuracy of step detection and energy expenditure estimation at different speeds for three research-grade accelerometers in overweight and obese participants under controlled laboratory settings.

## 2. Materials and Methods

Forty-eight overweight and obese subjects participated in this study (24 males). Subjects were on average 37.4 ± 13.9 years old, and their mean body mass index (BMI, kg/m^2^) was 31.4 ± 3.8; they were 173.6 ± 10.3 cm tall, weighted 94.8 ± 15.5 kg, had a skeletal muscle percentage (SMM%) of 36.9 ± 6.2, fat percentage of 34.4 ± 10.1 and waist circumference of 99.2 ± 12.0 cm (Table 1). Exclusion criteria for the participants were BMI less than 25 or over 40, younger than 20 or older than 75 years, any disease or injury preventing normal movement, arthritis, high blood pressure, chronic cardiovascular diseases, or acute cardiovascular event during the last year, ventilatory diseases and pregnancy or lactation. None of the subjects had undergone bariatric surgery for weight loss. This study was approved by the ethical committee in the Northern Ostrobothnia Hospital District (EETTMK 26/3/21). All the participants were healthy volunteers who gave their written informed consent in accordance with the Declaration of Helsinki. This study was conducted following national legislation, guidelines, and the Declaration of Helsinki.

All subjects participated in one measurement session conducted between 08:00 and 11:00 in the morning. Subjects were requested to fast at least 10 but not more than 16 h before their scheduled study session. They were also asked to avoid strenuous exercise on the day before and on the morning of the study session. Bioimpedance was used to determine the body composition (InBody 720, Biospace, Co, Ltd., Seoul, Korea). Energy expenditure was estimated via oxygen uptake and carbon dioxide production using indirect calorimetry (IC) (Vyntus CPX, Vyaire Medical GmbH, Hoechberg, Germany). Ergospirometer was calibrated every morning before the first subject and was considered valid for 4 h as instructed by the manufacturer. The contents of the gas were as follows: 5.0% CO_2_, 15.9 O_2,_ and the remaining 79.1% N_2_. Resting metabolic rate (RMR) was estimated in a supine position using a Hans Rudolph 7450 V2 mask (Hans Rudolph, Shawnee, Kansas, USA) until the values plateaued for at least 10 min and the last 5 min of the measurement were used to calculate the RMR. Respiratory exchange ratio (RER) was required to stay between 0.90 and 0.70 during the 10-min period. Weir equation was used to calculate the metabolic rate (kcal/day) = 1.44 (3.94VO_2_ + 1.11VCO_2_). RMR defined the level of 1 metabolic equivalent (MET) for the subsequent EE estimation analysis. 

After conducting the initial measurements, participants underwent an exercise routine on a treadmill (X-erfit 4000 Pro Run). The routine consisted of 6 speeds with a 4-min duration per speed with a total duration of 24 min. The speeds were 1.5, 3, 4.5, 6, 7.5, and 9 km/h. Acceleration to next speed took approximately 5 s at the beginning of each speed. A video camera was used to record participants’ feet during the entire exercise. The videos were used to count the actual step numbers at every speed and were performed according to Sushames et al., 2016 [22]. Energy expenditure was recorded during physical activity with a Hans Rudolph 7450 V2 mask. Energy expenditure for each speed was calculated using the Weir equation from the last minute of each speed and multiplying that with 4. Transformation to metabolic equivalents (METs) was performed using RMR as the level 1 MET.

Three accelerometers were worn by subjects during the exercise protocol. A Sartorio Xelometer (SX) (Sartorio Oy, Oulu, Finland) and an ActiGraph GT3X (AG) (ActiGraph LLC, Pensacola, FL, USA) were attached with elastic belts on the right side of the hip and an activPAL (AP) (PAL Technologies Ltd., Glasgow, Scotland) worn on the right thigh, all following the manufacturers’ recommendation. The data from the SX device was extracted using Sartorio software (v18) and detection algorithms provided by the manufacturer and were run on MATLAB R2019a for step counts, step intensities, and EE estimates (MET) [16]. For AP, PAL connect (v8.10.8.76) was used to set up the device and extract the data and PAL analysis (v8.11.2.54) to analyze the step counts and EE estimates (METhrs). The AP-derived MET-hours were transformed into METs. Finally, AG data was extracted with ActiLife (v6.13.4) and step counts were calculated using 1 s epochs and 100 Hz sampling rate. For EE (METs), Freedson Adult (1998) cut points were used (equation: MET rate = 1.439008 + (0.000795 × CPM) where CPM = counts per minute). In obese people, the mean amplitude deviation (MAD)—based method, such as the one in SX, provided the most accurate EE estimates (error-% 14.3) [23].

Mean absolute percentage errors (MAPEs) were calculated for every speed between the accelerometer-estimated step counts and actual steps (video) using the following equation:

$$M\%=\left(\frac{1}{n}\overset{n}{\underset{t=1}{\sum }}\left|\frac{A_{t}-F_{t}}{A_{t}}\right|\right)\times 100$$

Relevant disagreement was considered at MAPEs over 5%. EE data from the accelerometers and IC were analyzed as METs. To observe the similarity between methods, paired-samples *t*-tests, linear regression, and intraclass correlations (ICC) were calculated, and Bland–Altman plots generated. Paired samples *t*-tests were used to study the means of absolute values of observed and estimated measures (accelerometry vs. video, accelerometry vs. IC) and ICCs (Pearson) to study the reliability of the estimates. All statistical analyses were conducted, and figures generated using IBM SPSS Statistics v 26. *p*-values less than 0.05 were considered statistically significant. ICC over 0.90 was considered excellent, 0.75–0.90 good, 0.75–0.50 moderate, and less than 0.50 as poor. Results in the Tables are represented as mean ± standard deviation.

## 3. Results

### 3.1. Step Detection

**Figure 1:**
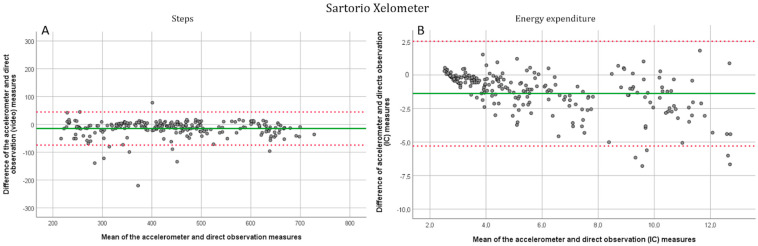
Bland-Altman plots for the Sartorio Xelometer. (**A**). Step detection compared to the direct (video) measurement. All six speeds have been plotted separately. (**B**). EE estimation compared with indirect (IC) calorimetry. Solid line marks the mean and dotted lines show the −1.96–1.96 SD limits.

**Figure 2:**
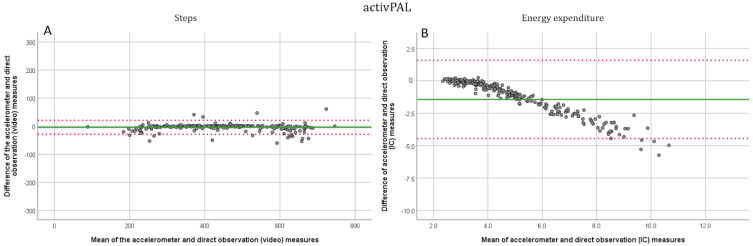
Bland-Altman plots for the activPAL. (**A**). Step detection compared to the direct (video) measurement. All six speeds have been plotted separately. (**B**). EE estimation compared with indirect (IC) calorimetry. Solid line marks the mean and dotted lines show the −1.96–1.96 SD limits.

**Figure 3:**
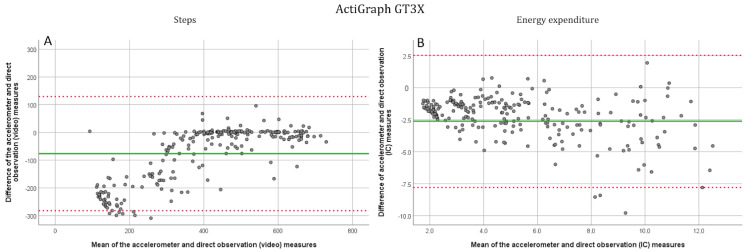
Bland-Altman plots for the ActiGraph GT3X. (**A**). Step detection compared to the direct (video) measurement. All six speeds have been plotted separately. (**B**). EE estimation compared with indirect (IC) calorimetry. Solid line marks the mean and dotted lines show the −1.96–1.96 SD limits.

All participants completed the three first speeds of the protocol, and one participant stopped after 4.5 km/h. A total of 38 out of 48 could complete the first running speed (7.5 km/h) and 25 completed the whole protocol. At walking speeds (1.5, 3, 4.5, and 6 km/h), the AP device was the most accurate. The corresponding MAPEs were 6.39, 0.95, 0.99, and 2.44, respectively (Table 2). For SX, the corresponding MAPEs were 9.73, 3.97, 2.91, and 6.28, respectively. The AG was the least accurate at the lower walking speeds but improved its accuracy from 4.5 km/h with MAPEs of 88.69, 31.50, 4.25, and 2.44, respectively. At the running speeds (7.5 and 9 km/h) the AG device performed better with MAPEs of 4.43 and 2.63, respectively. For the SX and the AP, the MAPEs were 2.26, 4.47, and 3.99, 5.18, respectively. The SX device estimate of the total number of steps differed by 3.48% and for the AP and the AG by 4.37% and 17.80%, respectively. Significant differences between direct measurement and accelerometer estimated steps were observed at 1.5 km/h and a first running speed (*p* < 0.000) for the AP. For the SX device, significant differences were observed at all speeds except 4.5 km/h and a first running speed (*p* < 0.05) between device estimates and direct observation, and for the AG at speeds of 1.5 and 3 km/h as well as in the first running speed (*p* < 0.000). The intraclass correlations were significant (*p* < 0.030) at all speeds for all devices except for the AG at 3 km/h (*p* = 0.646). For the AP device, the correlation coefficients were excellent (>0.90) and for the SX good or excellent (>0.75 and >0.90, respectively), except for the lowest speed where the correlation was moderate (0.61). For the AG, the correlation coefficients were excellent (>0.90) while running and brisk walking (6 km/h), good (>0.75) with the first running speed, and moderate or poor (>0.50, <0.50, respectively), at speeds of 1.5, 3 and 4.5. The R^2^ values for the regressions between actual and estimated steps were 0.948 for the SX, 0.963 for the AP, and 0.821 for the AG (Supplementary Materials). For the Bland–Altman plots the means, standard deviations, and 95% confidence intervals were −14.7 ± 30.3, upper 44.7, lower −74.2, respectively, for the SX (Figure 1A), −4.0 ± 12.8, upper 21.1, lower −29.0, respectively, for the AP (Figure 2A) and −76.39 ± 104.9, upper 129.3 and lower −282.0, respectively, for the AG (Figure 3A).

### 3.2. Energy Expenditure Estimation

All three devices were inaccurate in estimating energy expenditure (Table 3). The SX had the most accurate estimates of EE upon the complete exercise protocol with a MAPE of 18.43. The MAPEs for total EE were 49.62 for the AP and 36.16 for the AG with significant but poor intraclass correlations (*p* < 0.05, ICC < 0.50). For the SX, the speed-wise MAPEs from 1.5 km/h to the second running speed were 15.15, 17.60, 19.02, 21.41, 18.03, and 19.74. respectively. For the AP, the values were 12.36, 16.29, 27.82, 43.82, 56.10, 57.38 and for the ActiGraph 59.45, 40.67, 28.92, 29.88, 29.61 and 32.09, respectively. At all speeds for all three devices, there were significant differences between accelerometer estimates and indirect calorimetry (*p* ≤ 0.005), and no significant intraclass correlations were observed (*p* > 0.120). The R^2^ values for the regression between indirect calorimetry and device estimated EE (when considering all speeds together) were 0.81 for the SX, 0.75 for the AP, and 0.745 for the AG (Supplementary Materials). For the Bland–Altman plots the means, standard deviations, and 95% confidence intervals were for the SX (Figure 1B): −1.4 ± 2.0, upper 2.5, lower −5.3, respectively, for the AP (Figure 2B): −1.4 ± 1.5, upper 1.6, lower −4.5, respectively, and for the AG (Figure 3B): −2.6 ± 2.6, upper 2.5 and lower −7.8, respectively.

## 4. Discussion

We measured overweight and obese subjects without diseases or disabilities that could affect their gait. Forty-eight subjects performed an exercise protocol on a treadmill consisting of six different speeds, which were chosen to reflect the locomotion speeds of overweight, obese, and elderly people. The main objective was to study the accuracy of step detection and EE estimation with three known research accelerometers (SX, AP, and AG) in overweight and obese subjects. For step detection, similar accuracies for step detection were observed in this overweight/obese population as in normal weight subjects [18]. Energy expenditure estimates were inaccurately measured in all three devices. 

All three devices accurately estimated step detection when gait speed exceeded 4 km/h. Only the AG was inaccurate during slow walking speeds of 1.5 and 3 km/h with MAPE-% of 88.7 and 31.5, respectively. The AP showed the highest correlations between video camera-recorded steps and device step counts (ICC > 90). Step detection accuracy in overweight and obese people was similar compared to normal weight subjects with the exception that the AP was more accurate in estimating step counts during running in overweight and obese subjects [18]. Similar discrepancies have been reported by Feito and colleagues (2012) [20] who showed the increasing accuracy with increasing speed in the AG. Lee and colleagues [24] found a significant underestimation of step counts by AG at the speed of 3.2 km/h. We did not use the low-frequency extension for the AG data, since it has been shown to give indefinite results when applied to free-living data [25,26].

All accelerometers were inaccurate for estimating EE. The SX provided the smallest overall error percentages in the range of 15.15–21.41, while the AP and the AG ranged between 12.36–57.4 and 28.9–59.45, respectively. The accuracy of the AP EE estimation was at its highest during slow walking speeds (1.5 and 3 km/h) and decreased with speed. For the AG, an opposing trend was observed, the EE estimation accuracy was higher at speeds exceeding 4 km/h. For the SX, the EE estimation error was 12% smaller in overweight/obese subjects compared to normal weight subjects (MAPE 18.4 < 30.3) [18]. The opposite was observed with the AP and ActiGraph, both showing lower EE estimation accuracy with overweight subjects. The SX EE estimation is based on MAD and had the most accurate method of the three and is in line with the results of Diniz-Sousa and colleagues [23].

Applying accelerometry to overweight/obese populations is challenging. The excess body fat increases the energy used in bodily movements and can cause the accelerometer to be placed at an angle that has been shown to decrease accuracy [16]. If the manufacturer of the accelerometer has used a normal weighted population for algorithm development, inaccuracy will increase when applied to overweight people. The comparison of the different studies with objectively measured physical activity measures is problematic since the different manufacturers use their own methods and algorithms. Accelerations as g-values are further processed into steps, counts, and MET units for further analysis. Depending on the method, habitual daily PA can be classified differently into commonly used PA intensity classes such as light, moderate and vigorous [9]. Considering these points together with the discrepancies concerning wear location, time, signal processing, and filtering [8], a standardized method of measurement is needed to create accurate, specified, and personalized PA recommendations.

Our study is the first to evaluate the accuracy of EE estimation of these accelerometers at realistic walking and running speeds in overweight and obese subjects. The use of a video camera to record true step numbers, the use of both sexes, and a wide range of ages and BMIs are the strengths of this study. Our limitations include the lack of self-selected locomotion speed and the exclusion of any wrist-worn accelerometers. The gait speeds chosen are sufficient in covering the spectrum of overweight human locomotion speeds. Our results will guide users studying physical activity in different populations in the interpretation of their results and their conclusions towards public health recommendations.

## Figures and Tables

**Table 1:** Characteristics of the study population. SMM = skeletal muscle mass.

Sex	24 Male, 24 Female	Min.–Max.
Age (years)	37.4 ± 14.1	21–74
Height (cm)	173.6 ± 10.3	153.5–194.0
Weight (kg)	94.8 ± 15.5	70.2–142.5
BMI	31.4 ± 3.8	26.5–39.7
SMM-% (impedance)	36.9 ± 6.2	27.3–51.1
Fat-% (impedance)	34.4 ± 10.1	12.2–50.9
Waist circumference (cm)	99.2 ± 12.0	82.0–133.0

**Table 2:** Step detection statistics. Mean absolute percentage error (MAPE), paired sample *t*-test statistics with mean ± SD, lower and upper limits for 95% confidence intervals and *p*-values, and intraclass correlation (ICC) statistics with 95% CI presented for every accelerometer at separate speeds and for total duration of exercise protocol. * Shows statistical significance.

STEPS			Paired Samples *t*-Test	95% Confidence Interval of the Difference			ICC	95% Confidence Interval		F Test	
Sartorio	Speed (km/h)	MAPE-% ± Std. Dev.	Mean ± Std. Dev.	Lower	Upper	Sig. (2-Tailed)		Lower	Upper	Value	Sig.
	1.5	9.73 ± 7.82	19.68 ± 32.10	9.55	29.81	0.000 *	0.61	0.27	0.79	2.56	0.002 *
	3	3.97 ± 7.48	10.05 ± 30.41	0.45	19.65	0.041 *	0.84	0.71	0.92	6.39	0.000 *
	4.5	2.91 ± 3.35	2.59 ± 17.56	−2.96	8.13	0.351	0.93	0.86	0.96	13.85	0.000 *
	6	6.28 ± 8.02	28.87 ± 41.85	15.49	42.26	0.000 *	0.79	0.59	0.89	4.67	0.000 *
	Run1	2.26 ± 1.46	4.43 ± 16.02	−1.070	9.93	0.111	0.99	0.97	0.99	74.43	0.000 *
	Run2	4.47 ± 3.08	26.54 ± 24.35	16.26	36.82	0.000 *	0.98	0.96	0.99	62.47	0.000 *
	Total	3.48 ± 3.03	82.02 ± 75.94	58.05	105.99	0.000 *	0.90	0.81	0.94	9.81	0.000 *
activPAL											
	1.5	6.39 ± 8.10	14.90 ± 23.79	7.74	22.04	0.000 *	0.94	0.88	0.96	15.65	0.000 *
	3	0.95 ± 1.59	1.60 ± 7.17	−0.55	3.75	0.141	0.99	0.99	1.00	135.88	0.000 *
	4.5	0.99 ± 2.75	−0.29 ± 10.94	−3.58	3.00	0.860	0.98	0.96	0.99	41.68	0.000 *
	6	2.44 ± 5.45	−1.42 ± 22.75	−8.26	5.41	0.677	0.98	0.97	0.99	62.54	0.000 *
	Run1	3.99 ± 5.25	22.68 ± 33.36	11.55	33.80	0.000 *	0.94	0.88	0.97	16.19	0.000 *
	Run2	5.18 ± 4.60	17.39 ± 43.05	−1.22	36.00	0.066	0.91	0.8	0.96	11.68	0.000 *
	Total	4.37 ± 10.53	42.27 ± 213.67	−21.93	106.46	0.191	0.95	0.91	0.97	19.49	0.000 *
ActiGraph											
	1.5	88.69 ± 10.93	242.35 ± 47.32	228.30	256.40	0.000 *	0.44	−0.018	0.69	1.77	0.029 *
	3	31.50 ± 18.87	119.85 ± 82.36	95.39	144.31	0.000 *	−0.12	−1.02	0.38	0.89	0.646
	4.5	4.25 ± 9.11	13.37 ± 45.19	−0.05	26.79	0.051	0.58	0.25	0.77	2.40	0.002 *
	6	5.23 ± 9.35	11.59 ± 43.62	−1.37	24.54	0.078	0.94	0.89	0.97	17.14	0.000 *
	Run1	4.43 ± 10.3	19.50 ± 68.82	−3.12	42.12	0.089	0.80	0.61	0.89	4.91	0.000 *
	Run2	2.63 ± 1.56	12.72 ± 16.28	6.00	19.44	0.000 *	0.99	0.98	1.00	142.15	0.000 *
	Total	17.80 ± 9.48	381.35 ± 221.25	315.65	447.05	0.000 *	0.95	0.92	0.97	21.61	0.000 *

**Table 3:** Energy expenditure (EE) estimation statistics. Mean absolute percentage error (MAPE), paired sample *t*-test statistics with mean ± SD, lower and upper limits for 95% confidence intervals and *p*-values and intraclass correlation (ICC) statistics with 95% CI presented for every accelerometer at separate speeds and for total duration of exercise protocol. * Shows statistical significance.

MET			Paired Samples *t*-Test	95% Confidence Interval of the Difference			ICC	95% Confidence Interval		F Test	
Sartorio	Speed (km/h)	MAPE-% ± Std. Dev.	Mean ± Std. Dev.	Lower	Upper	Sig. (2-Tailed)		Lower	Upper	Value	Sig.
	1.5	15.15 ± 13.72	0.37 ± 0.81	0.11	0.62	0.005 *	0.11	−0.67	0.53	1.12	0.353
	3	17.60 ± 13.16	0.73 ± 0.96	0.42	1.04	0.000 *	0.21	−0.47	0.58	1.27	0.223
	4.5	19.02 ± 11.75	1.04 ± 1.05	0.71	1.38	0.000 *	0.23	−0.43	0.59	1.31	0.199
	6	21.41 ± 12.92	1.74 ± 1.59	1.21	2.25	0.000 *	0.21	−0.51	0.58	1.26	0.237
	Run1	18.03 ± 12.21	1.83 ± 2.07	1.09	2.57	0.000 *	0.18	−0.64	0.59	1.22	0.282
	Run2	19.74 ± 11.89	2.59 ± 2.11	1.64	3.52	0.000 *	0.08	−1.19	0.62	1.09	0.417
	Total	18.43 ± 13.59	1.31 ± 1.37	0.86	1.74	0.000 *	0.28	−0.34	0.62	1.40	0.146
activPAL											
	1.5	12.36 ± 11.20	0.30 ± 0.68	0.09	0.51	0.005 *	0.37	−0.16	0.65	1.58	0.068
	3	16.29 ± 12.65	0.74 ± 0.81	0.49	0.99	0.000 *	0.29	−0.3	0.61	1.41	0.134
	4.5	27.82 ± 11.92	1.60 ± 0.97	1.30	1.90	0.000 *	0.17	−0.51	0.55	1.21	0.264
	6	43.82 ± 10.70	3.48 ± 1.61	2.95	3.99	0.000 *	0.05	−0.79	0.50	1.06	0.425
	Run1	56.10 ± 7.43	6.18 ± 1.93	5.48	6.87	0.000 *	−0.03	−1.12	0.49	0.96	0.538
	Run2	57.38 ± 6.40	7.39 ± 1.91	6.49	8.28	0.000 *	0.13	−1.18	0.65	1.15	0.378
	Total	49.62 ± 11.21	3.37 ± 1.25	2.97	3.75	0.000 *	0.49	0.07	0.72	1.99	0.013 *
ActiGraph											
	1.5	59.45 ± 9.40	1.95 ± 0.74	1.72	2.17	0.000 *	0.15	−0.55	0.53	1.17	0.295
	3	40.67 ± 14.07	1.82 ± 1.00	1.51	2.12	0.000 *	−0.10	−1.03	0.39	0.90	0.631
	4.5	28.92 ± 13.17	1.61 ± 1.13	1.26	1.95	0.000 *	0.17	−0.5	0.55	1.21	0.260
	6	29.88 ± 15.19	2.37 ± 1.66	1.84	2.89	0.000 *	0.30	−0.29	0.63	1.44	0.124
	Run1	29.61 ± 17.13	3.16 ± 2.51	2.25	4.06	0.000 *	0.27	−0.49	0.64	1.37	0.190
	Run2	32.09 ± 16.50	4.20 ± 2.69	3.00	5.39	0.000 *	−0.34	−2.23	0.44	0.74	0.746
	Total	36.16 ± 15.06	2.42 ± 1.41	1.98	2.84	0.000 *	0.43	−0.03	0.69	1.77	0.031 *

## Supplementary Materials

The following supporting information can be downloaded at: https://www.mdpi.com/article/10.3390/jcm11123267/s1. Figure S1. Regression plots for Sartorio Xelometer. Figure S2. Regression plots for activPAL. Figure S3. Regression plots for ActiGraph GT3X.
