# Supplementary material for: Step Detection Accuracy and Energy Expenditure Estimation at Different Speeds by Three Accelerometers in a Controlled Environment in Overweight/Obese Subjects
Source: J Clin Med. 2022 Jun 7;11(12):3267. doi: 10.3390/jcm11123267 (PMC9224826; doi:10.3390/jcm11123267)
Supplement: Supplementary file 1 [file jcm-11-03267-s001.zip › jcm-1734285-supplementary.pdf]

Step detection accuracy and energy expenditure estimation at different speeds by three accelerometers in a controlled environment in overweight/obese subjects

Ville Stenbäck[1], Juhani Leppäluoto[1], Rosanna Juustila[1], Laura Niiranen[1], Dominique Gagnon[2,3,4,5], Mikko Tulppo[1] and Karl-Heinz Herzig [1,6,*]

Affiliations:

[1]Research Unit of Biomedicine, Medical Research Center, Faculty of Medicine, University of Oulu, Oulu University Hospital, Oulu 90220, Finland

[2]Helsinki Clinic for Sports and Exercise Medicine, Foundation for Sports and Exercise Medicine, Helsinki 00550, Finland.

[3]Department of Sports and Exercise Medicine, Clinicum, University of Helsinki, Helsinki 00014, Finland

[4]School of Kinesiology and Health Sciences, Laurentian University, Sudbury P3E2C6, Canada

[5]Center for Research in Occupational Safety and Health, Laurentian University, Sudbury P3E2C6, Canada

[6]Department of Pediatric Gastroenterology and Metabolic Diseases, Poznan University of Medical Sciences, Poznan 61-701, Poland.

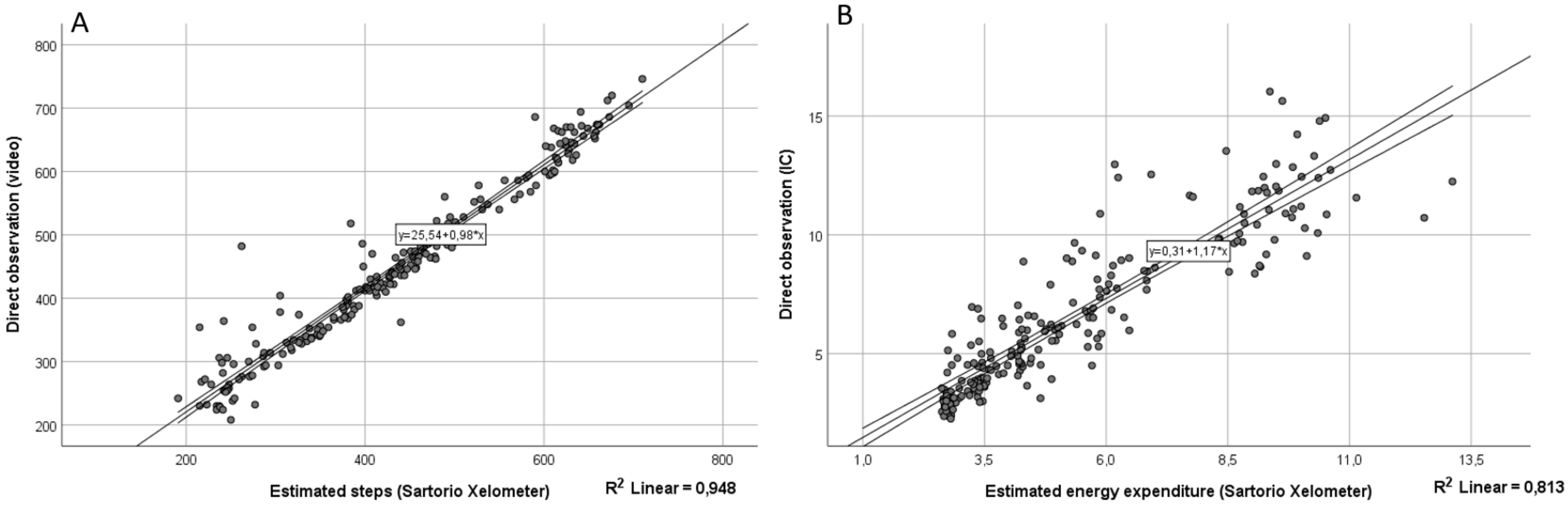

Supplementary Figure S1. Regression plots for Sartorio Xelometer. A. The regression plot between accelerometer estimated step counts and direct measurement (video). B. The regression plot between accelerometer estimated EE and direct measurement. All six speeds have been plotted separately.

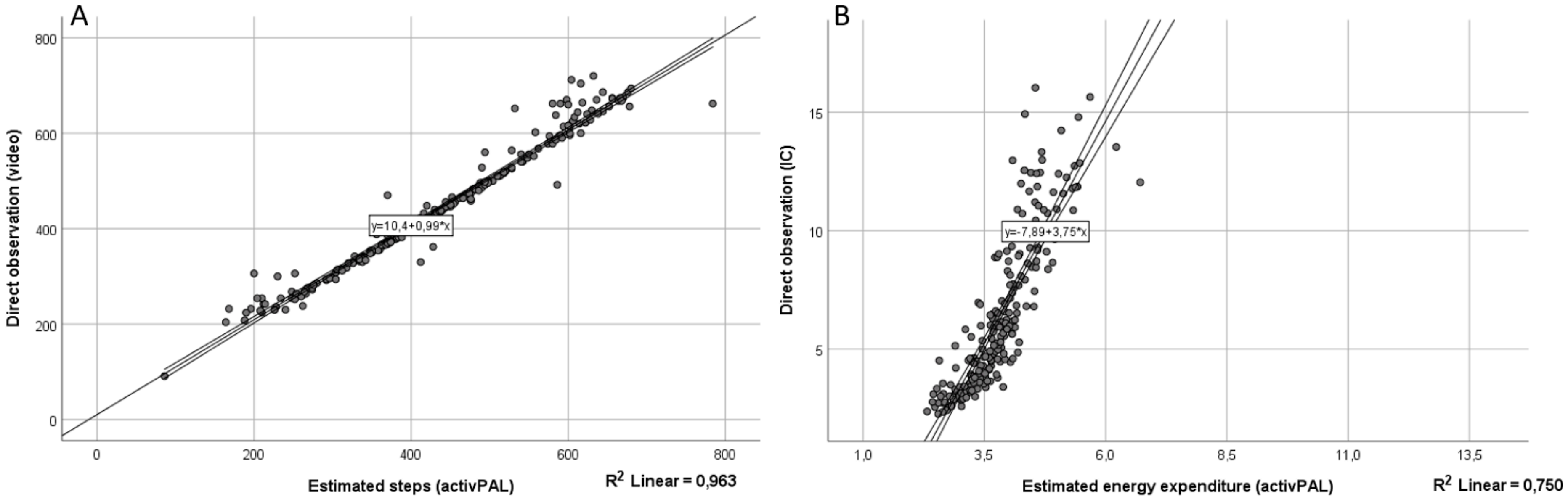

Supplementary Figure S2. Regression plots for activPAL. A. The regression plot between accelerometer estimated step counts and direct measurement (video). B. The regression plot between accelerometer estimated EE and direct measurement. All six speeds have been plotted separately.

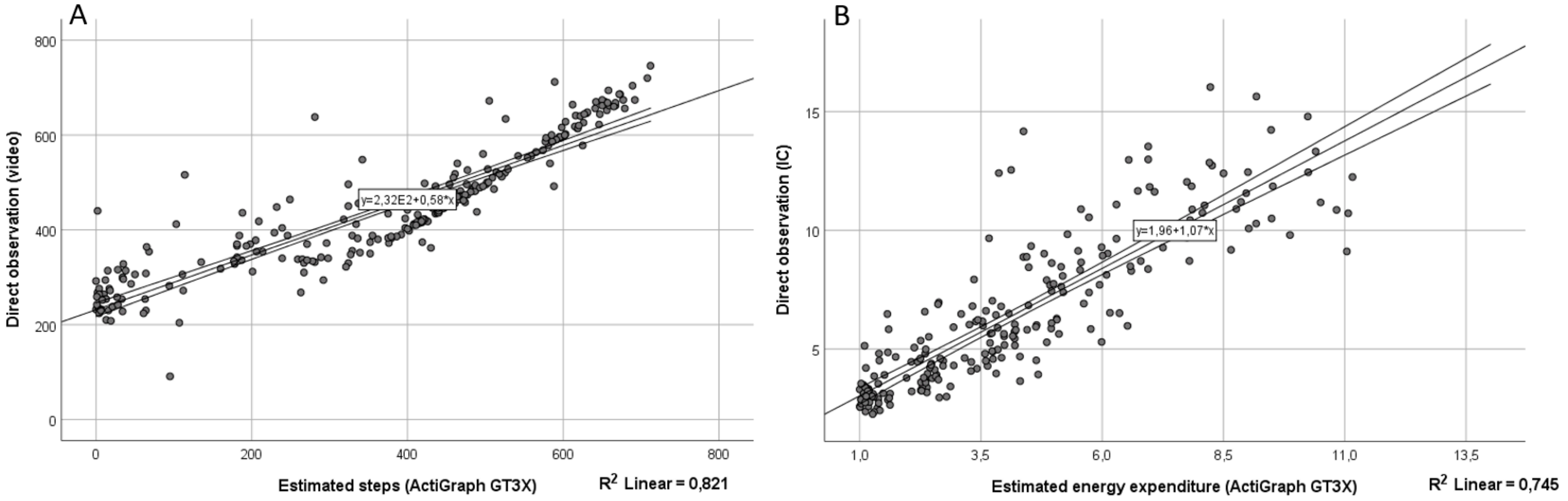

Supplementary Figure S3. Regression plots for ActiGraph GT3X. A. The regression plot between accelerometer estimated step counts and direct measurement (video). B. The regression plot between accelerometer estimated EE and direct measurement. All six speeds have been plotted separately.
